# Supplementary material for: Genome-wide association studies for earliness, MYMIV resistance, and other associated traits in mungbean (Vigna radiata L. Wilczek) using genotyping by sequencing approach
Source: PeerJ. 2024 Jan 26;12:e16653. doi: 10.7717/peerj.16653 (PMC10823994; doi:10.7717/peerj.16653)
Supplement: Supplemental Information 3 [file peerj-12-16653-s003.pdf]

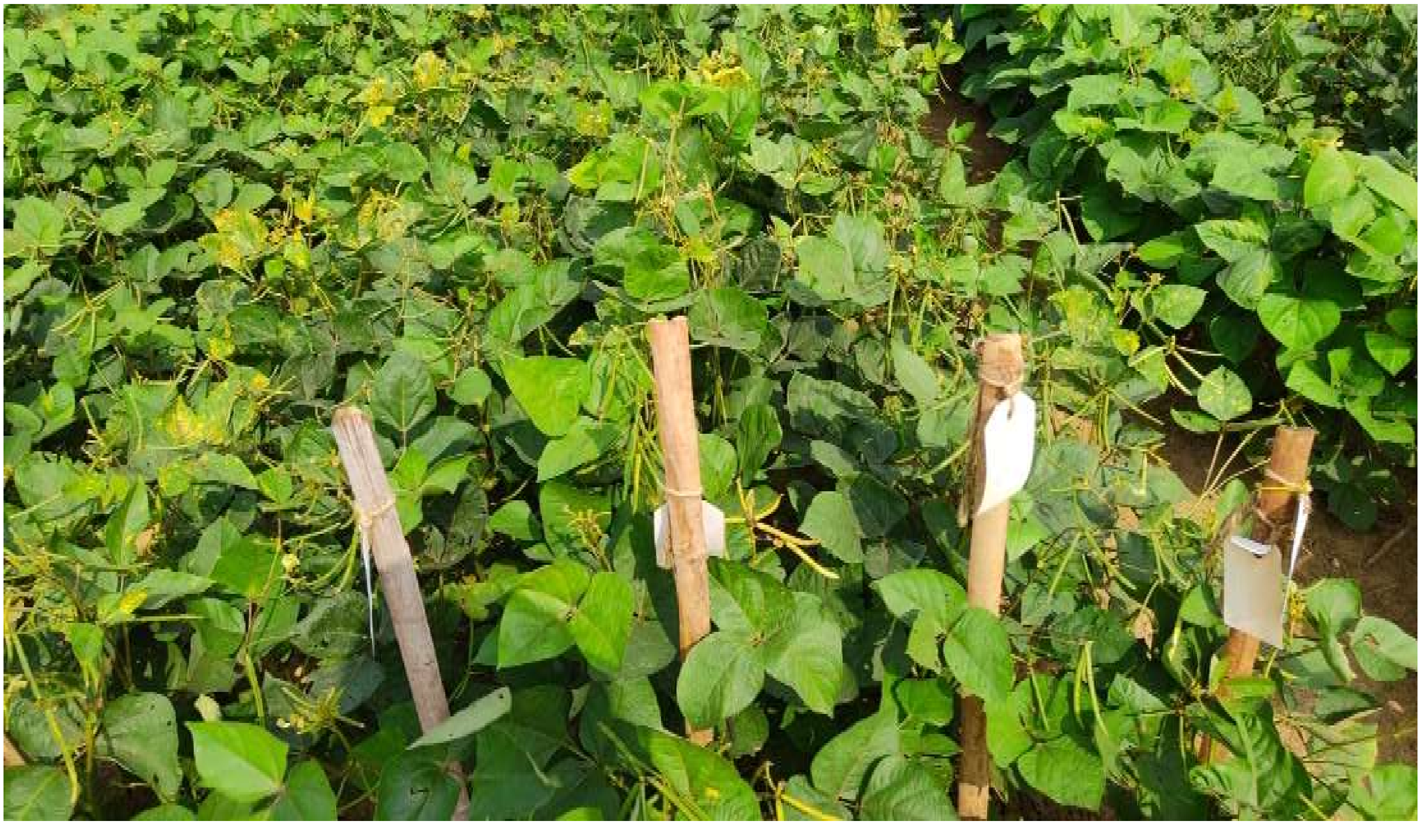

Figure S1. Mungbean genotypes grown in field.

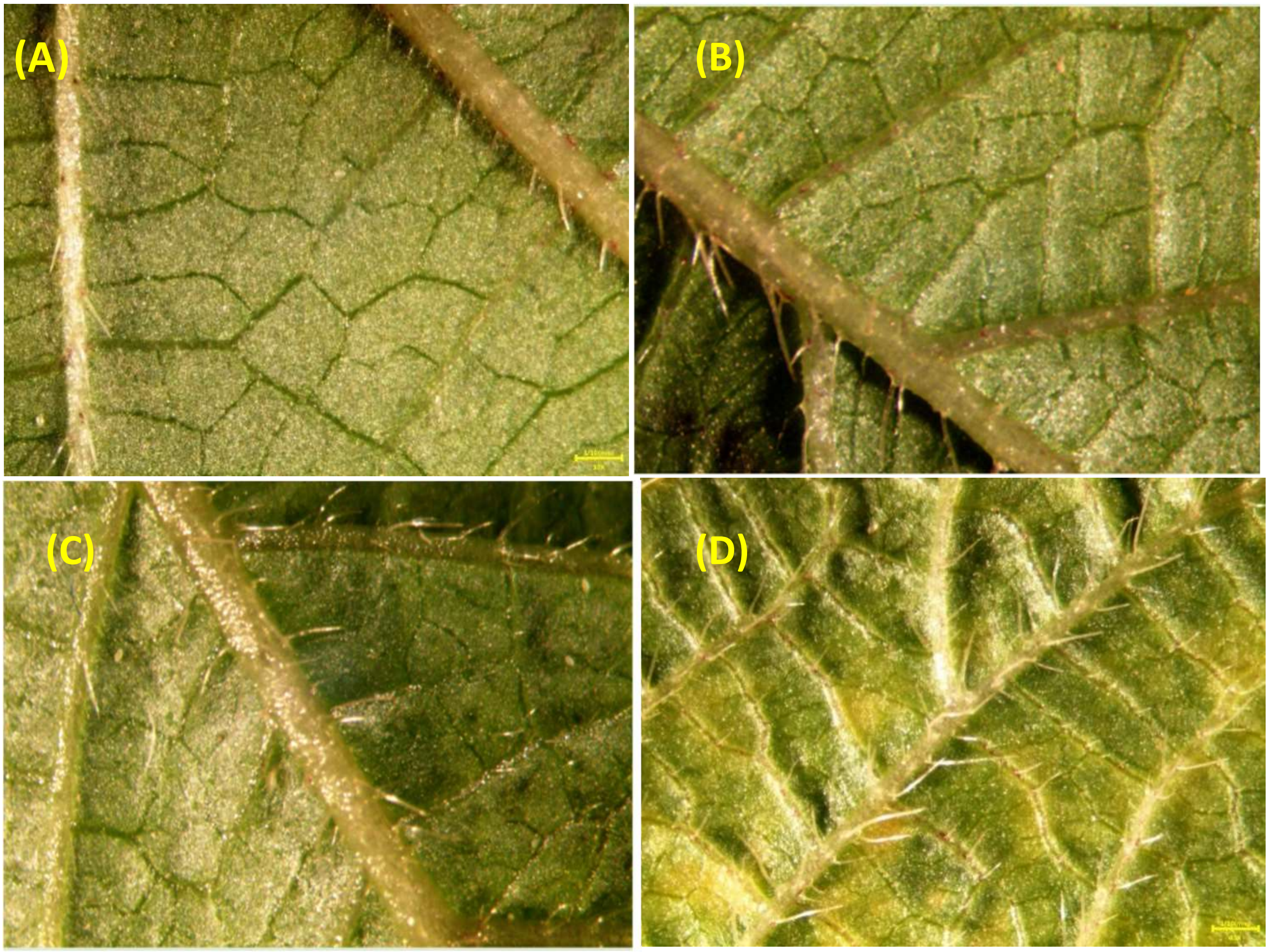

Figure S2. Trichomes range in different scale. (A) scale range 1, (B) scale range 2, (C) scale range 3, (D) scale range 4.

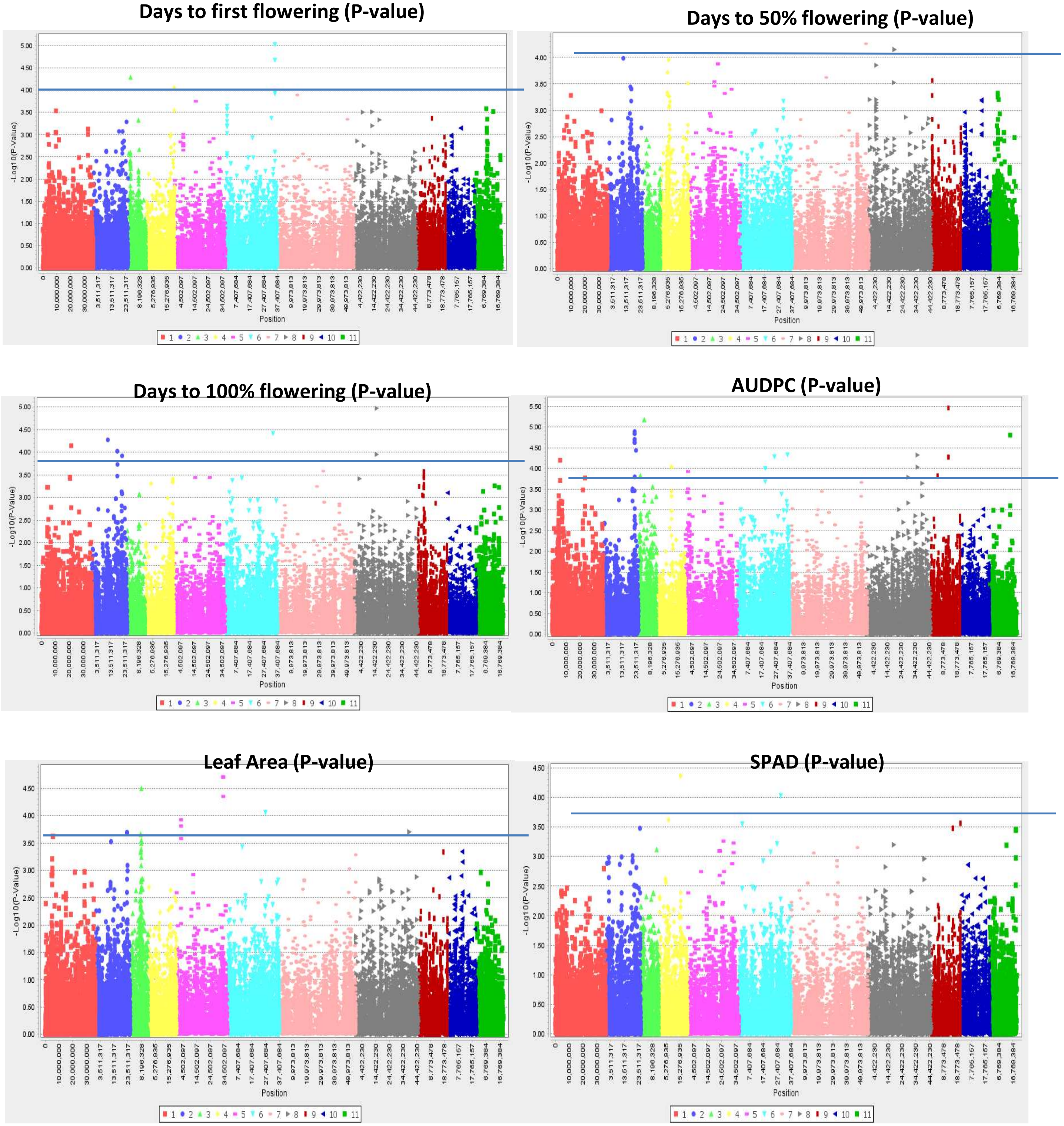

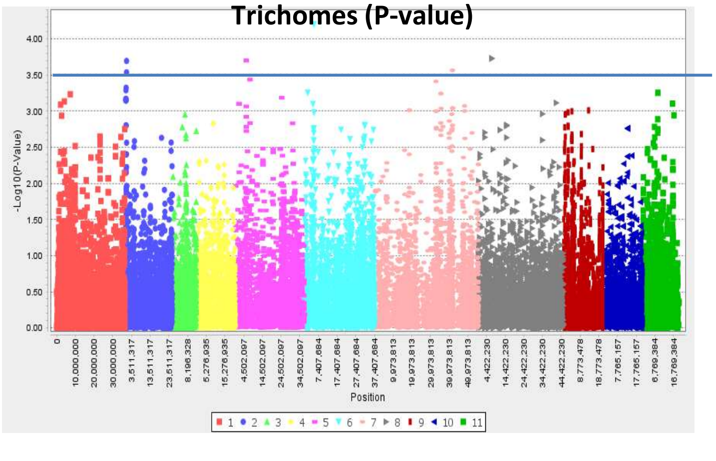

Figure S3. Manhattan plots showing significant p-values (GLM) for earliness and YMD traits. Colors indicate different chromosomes while horizontal lines indicate a common significance level of p=0.0001 [-log(p)=4.0]. Traits: Days to first flowering, days to 50% flowering, days to 100% flowering, AUDPC, Leaf area, SPAD value & Trichomes.

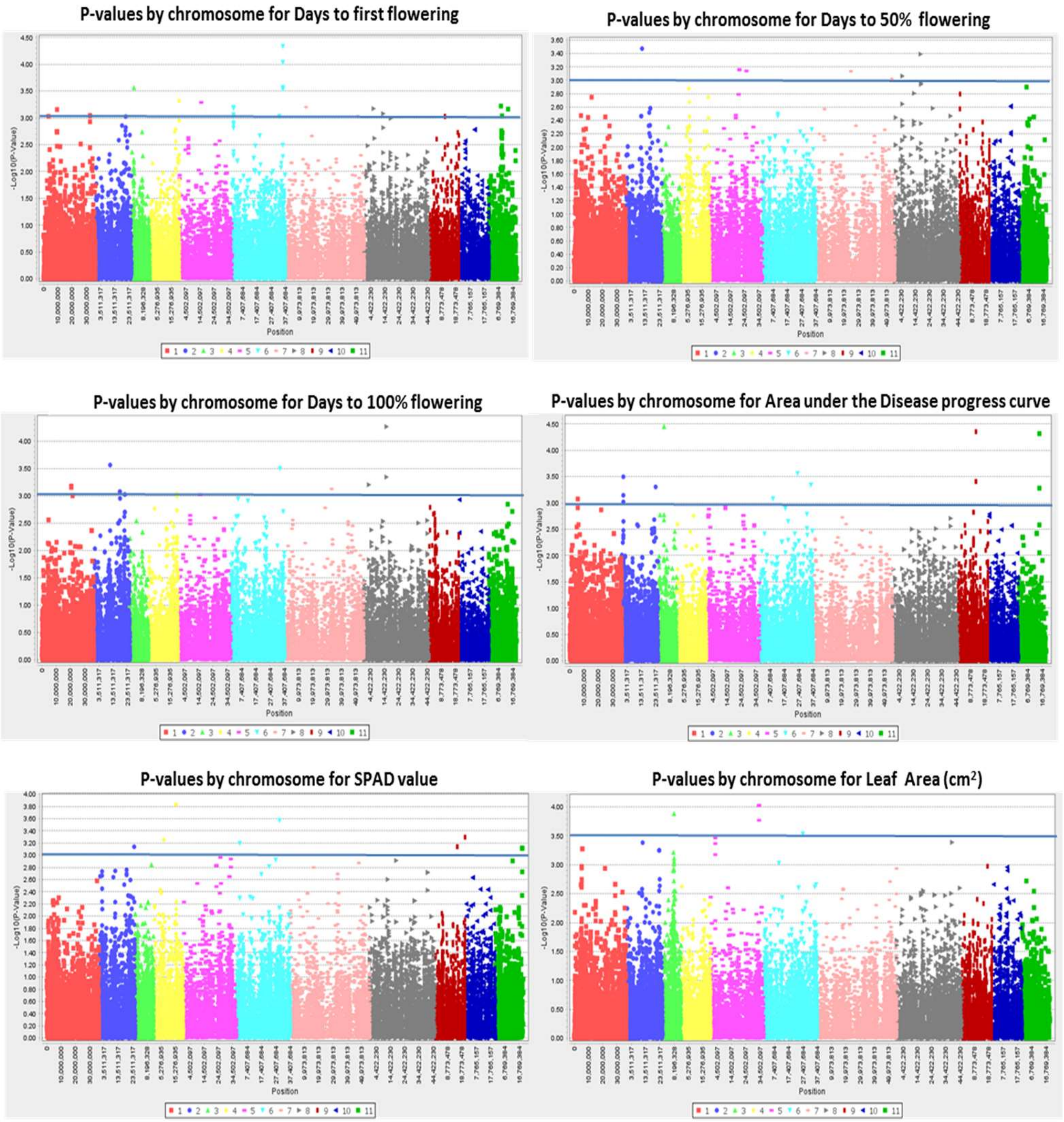

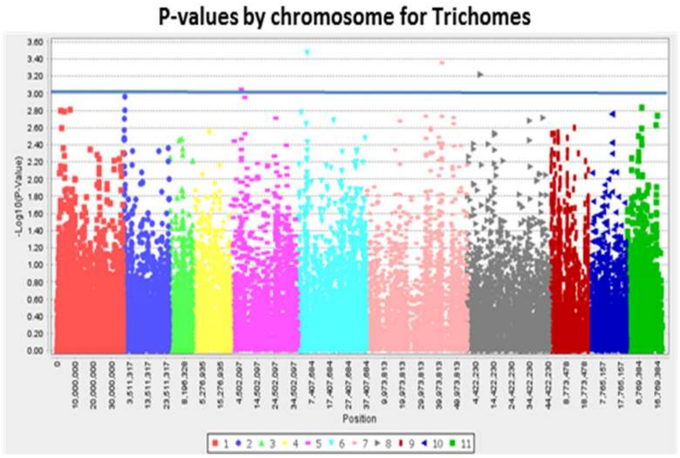

Figure S4. Manhattan plots showing significant p- values (MLM) for earliness and YMD traits. Colors indicate chromosomes while horizontal lines indicate a common significance level of p = 0.0001 [-log(p) =4.0 & 3.0]. Traits: Days to first flowering, days to 50% flowering, days to 100% flowering, AUDPC, Leaf area, SPAD value & trichomes.

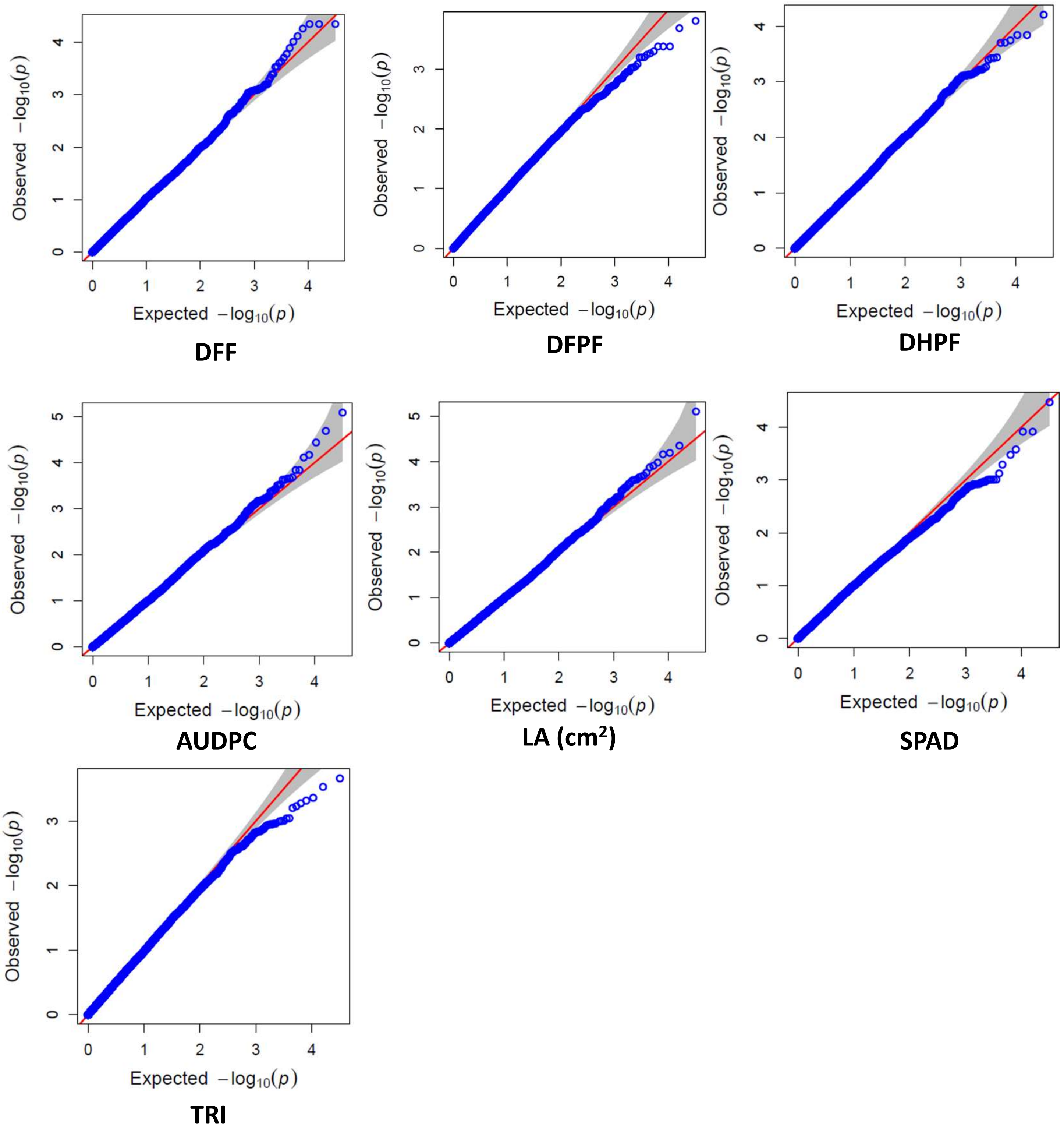

Figure S5. Quantile Quantile plots illustrating the comparison between expected and observed –log10(p)-values to detect significant genomic loci (genes) associated with earliness and MYMIV disease related traits in mungbean. *DFF, days to first flowering; DFPF, days to 50% flowering; DHPF, days to 100% flowering; AUDPC, Area under the disease progressive curve; LA, leaf area; SPAD, Soil Plant Analysis Development; TRI, trichomes;.*

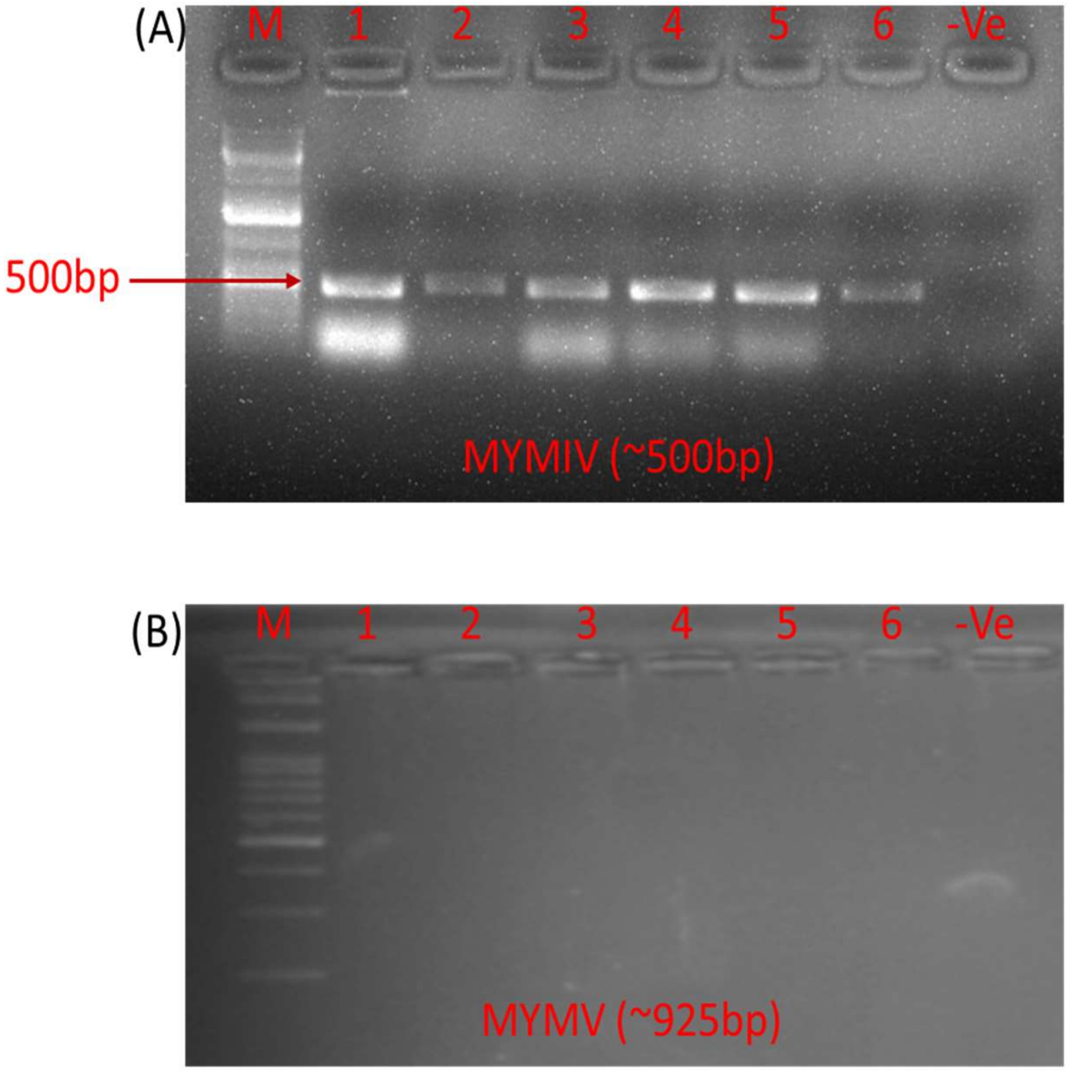

Figure S6: MYMIV gene specification amplification of susceptible mungbean genotypes. (A) MYMIV specific amplification (~500bp) using AV1 gene specific primers (BM925F and BM926R). The presence of band was observed in all the susceptible mungbean samples. B) No amplification was observed when MYMV gene specific primers (AVI1- Fwd and AC1- Rev) was used. Where, M: DNA ladder; -Ve: Negative control; 1-6: No. of susceptible mungbean genotypes; M: Marker

Days to Flowering

(a)

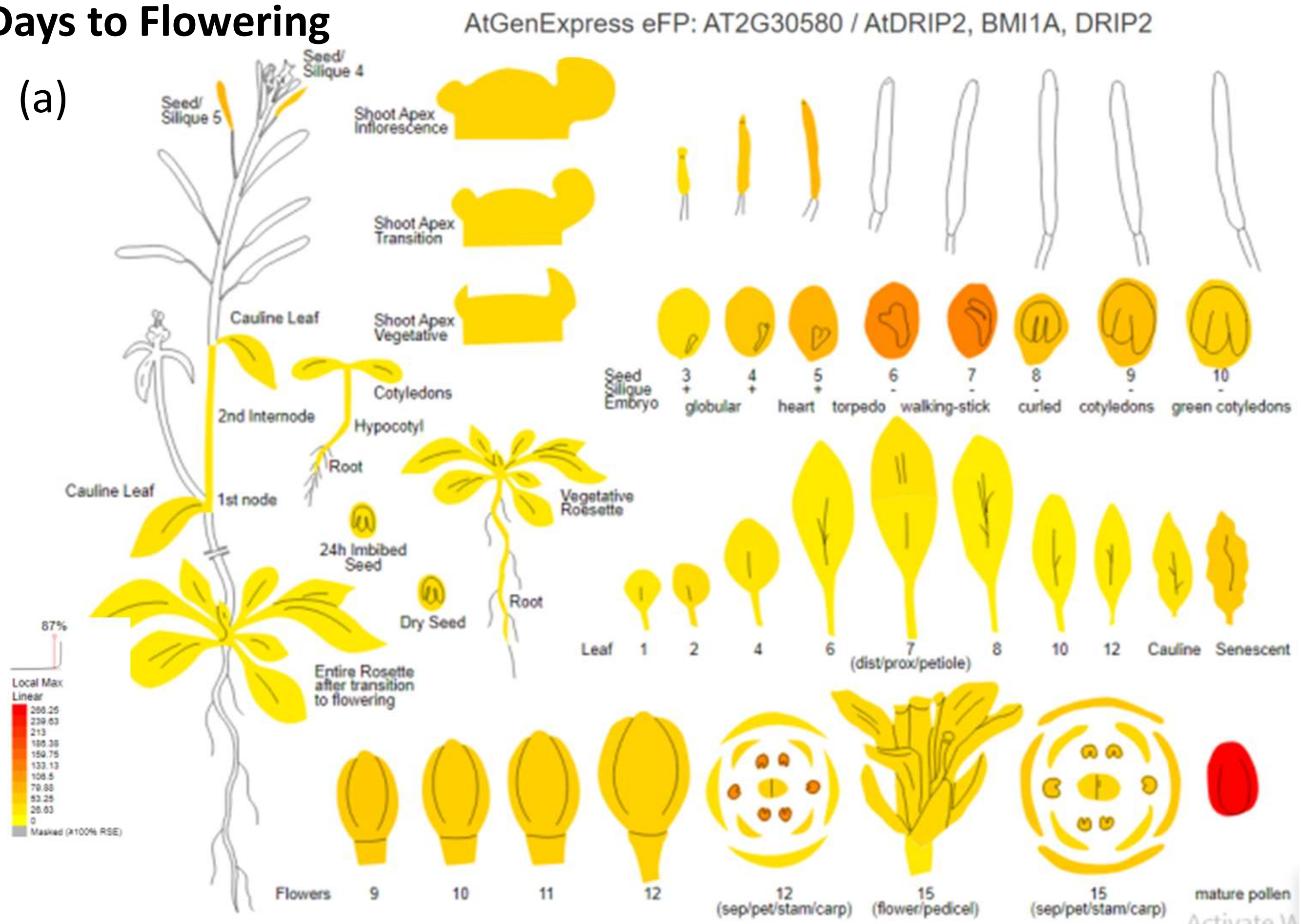

E3 ubiquitin protein ligase DRIP2

(b)

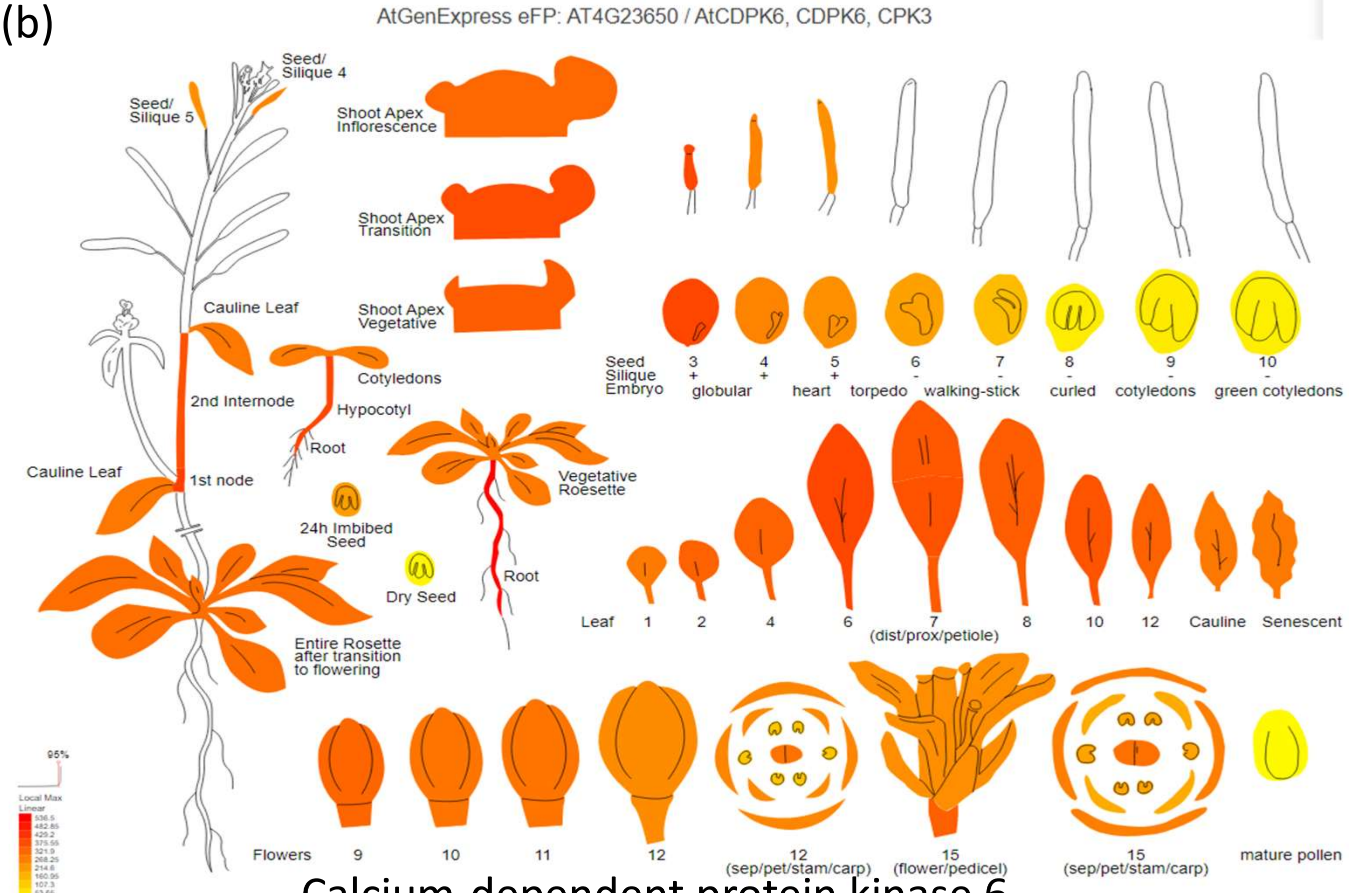

Calcium-dependent protein kinase 6

Figure S7. Details of 11 candidate genes validated using in-silico expression analysis. **(a)** E3 ubiquitin protein ligase DRIP2 expression for flowering on chr. 6. **(b)** Calcium-dependent protein Kinase 1 expression for flowering on chr. 7.

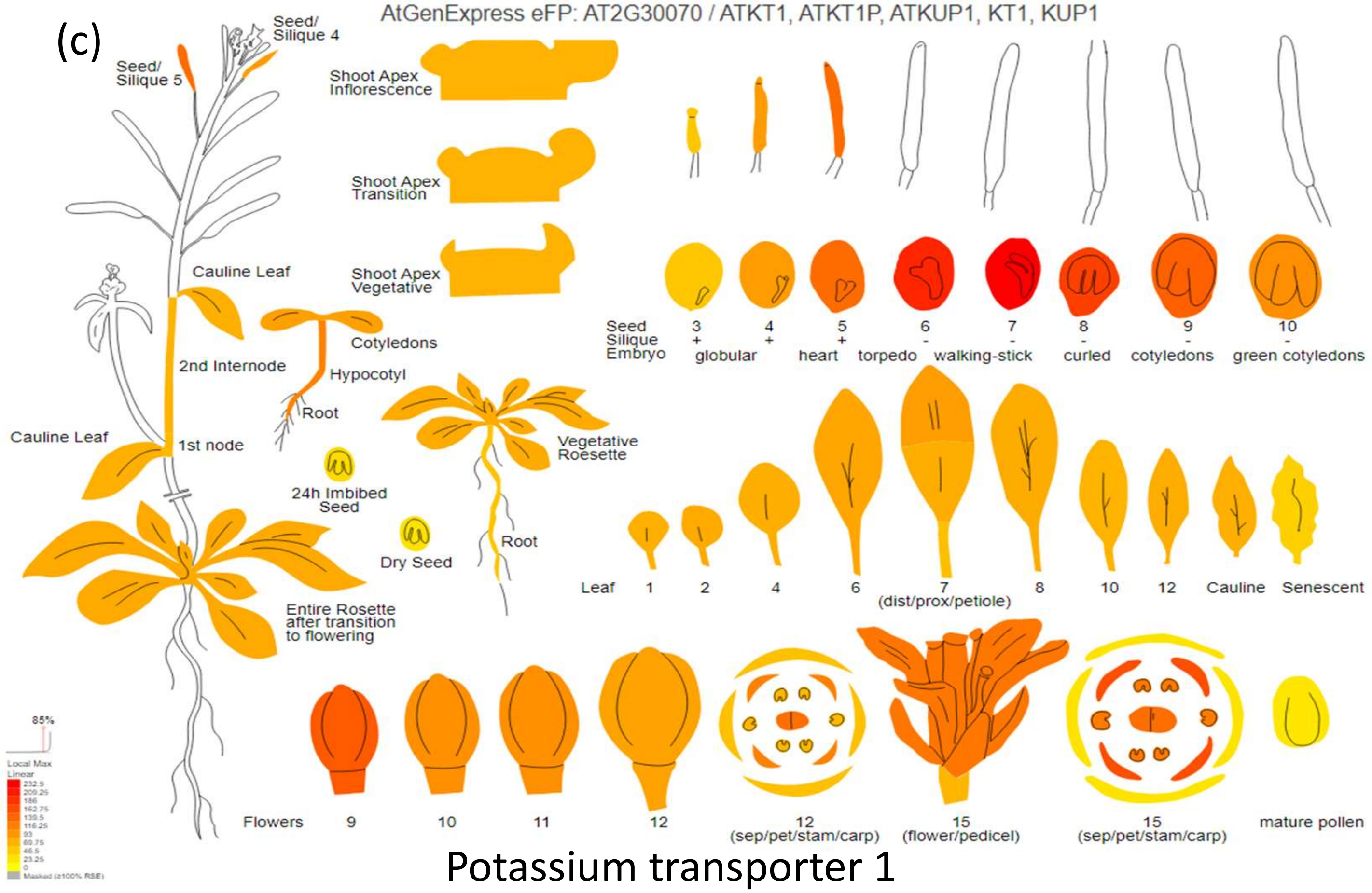

Potassium transporter 1

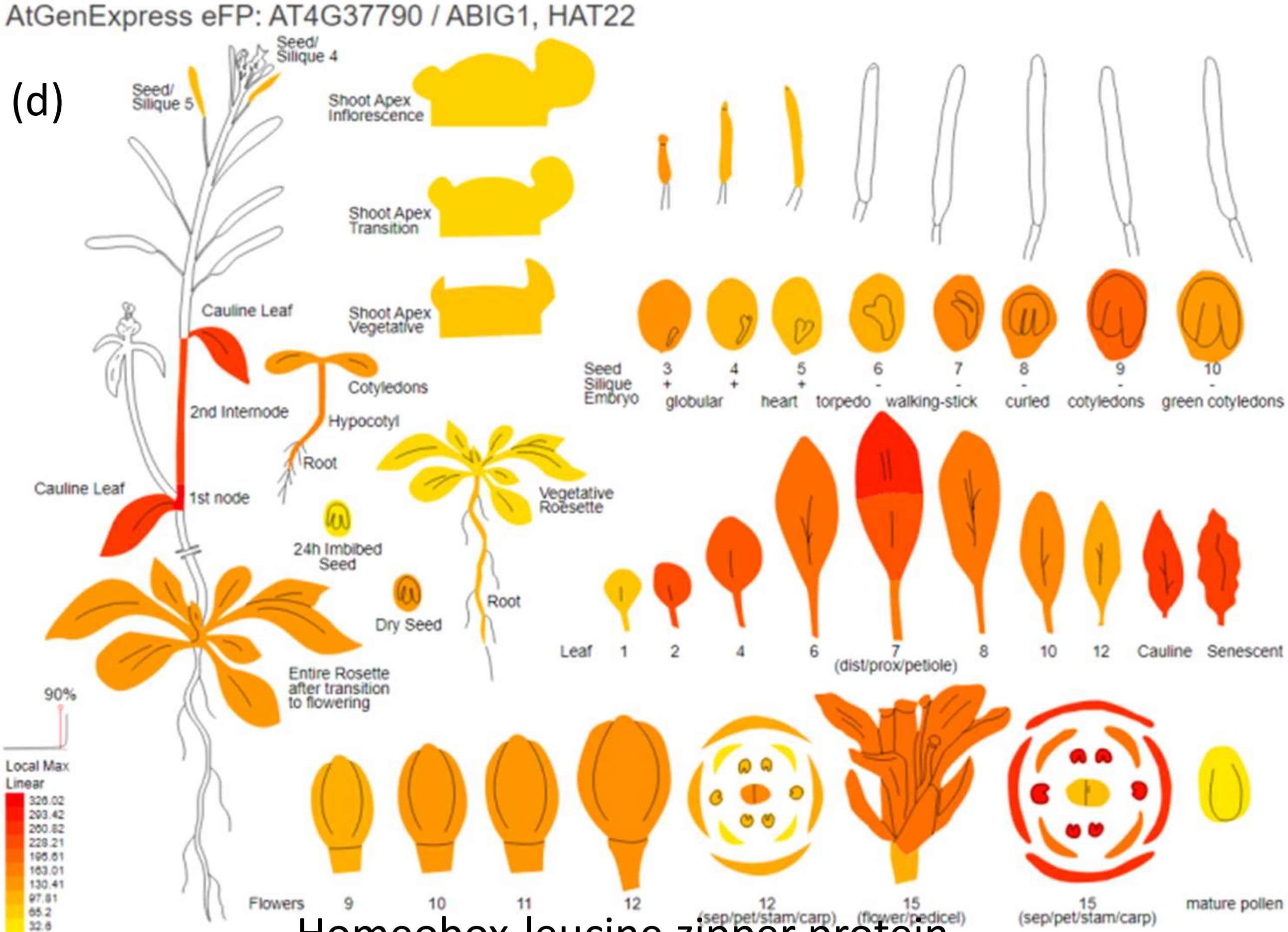

Homeobox-leucine zipper protein

Figure S7. (c). Potassium transporter 1 expression for flowering on chr. 7. **(d)** Homeobox-leucine zipper protein expression for flowering on chr. 5.

Disease related

(e)

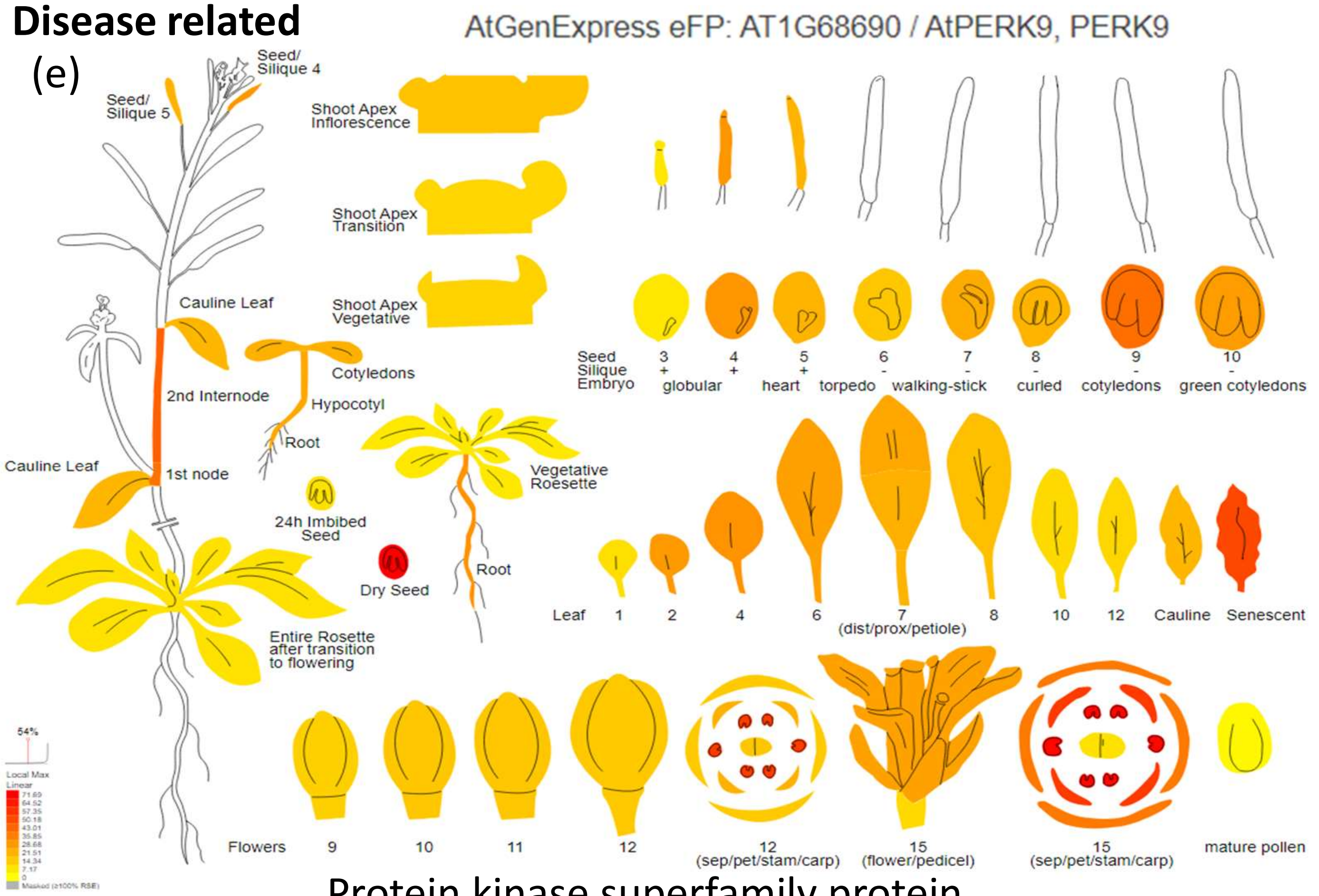

Protein kinase superfamily protein

(f)

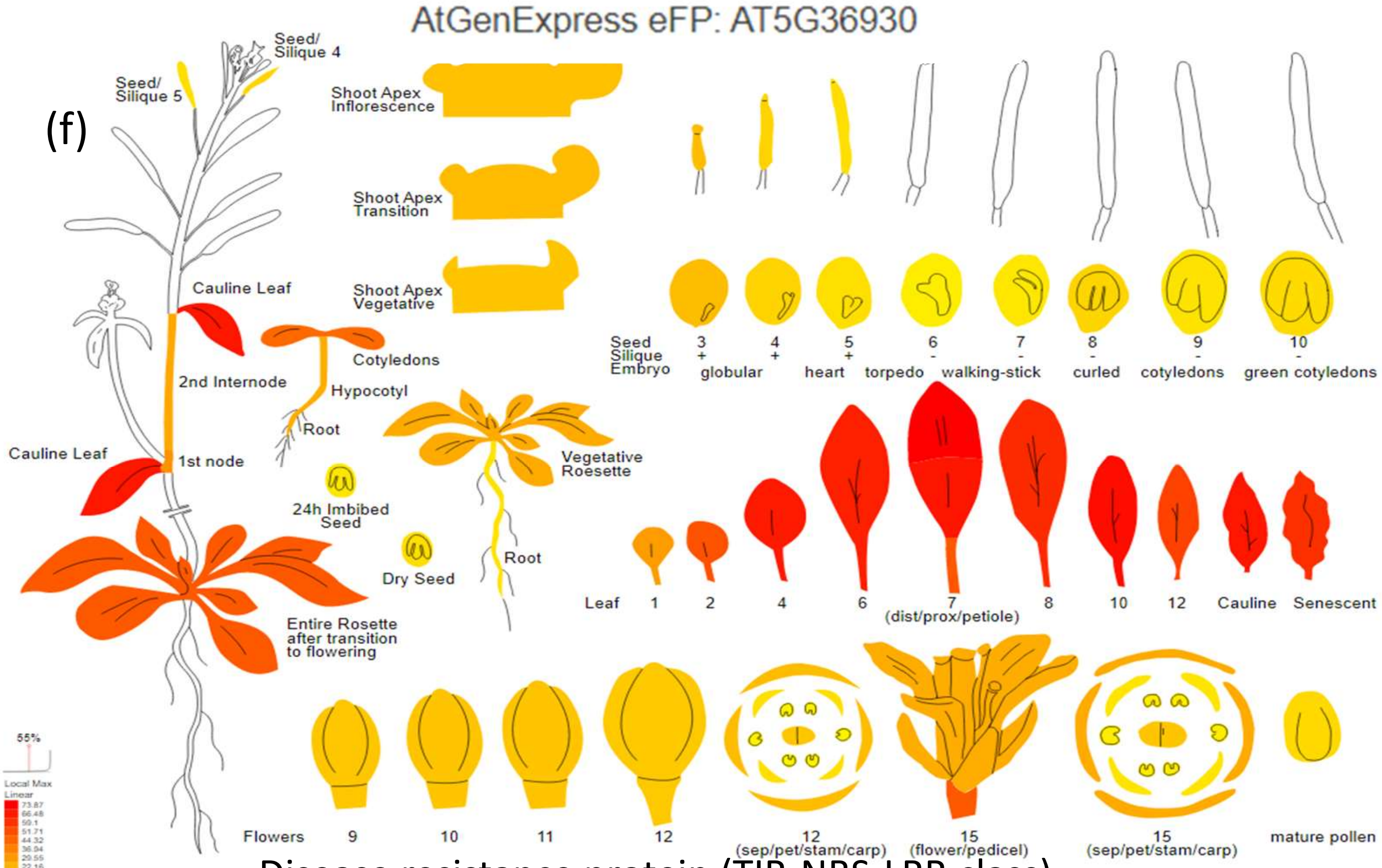

Disease resistance protein (TIR-NBS-LRR class)

Figure S7. (e) Protein kinase superfamily protein expression for disease resistance in leaves on chr. 6. (f) Disease resistance protein (TIR-NBS-LRR class) expression for YMD on chr. 9.

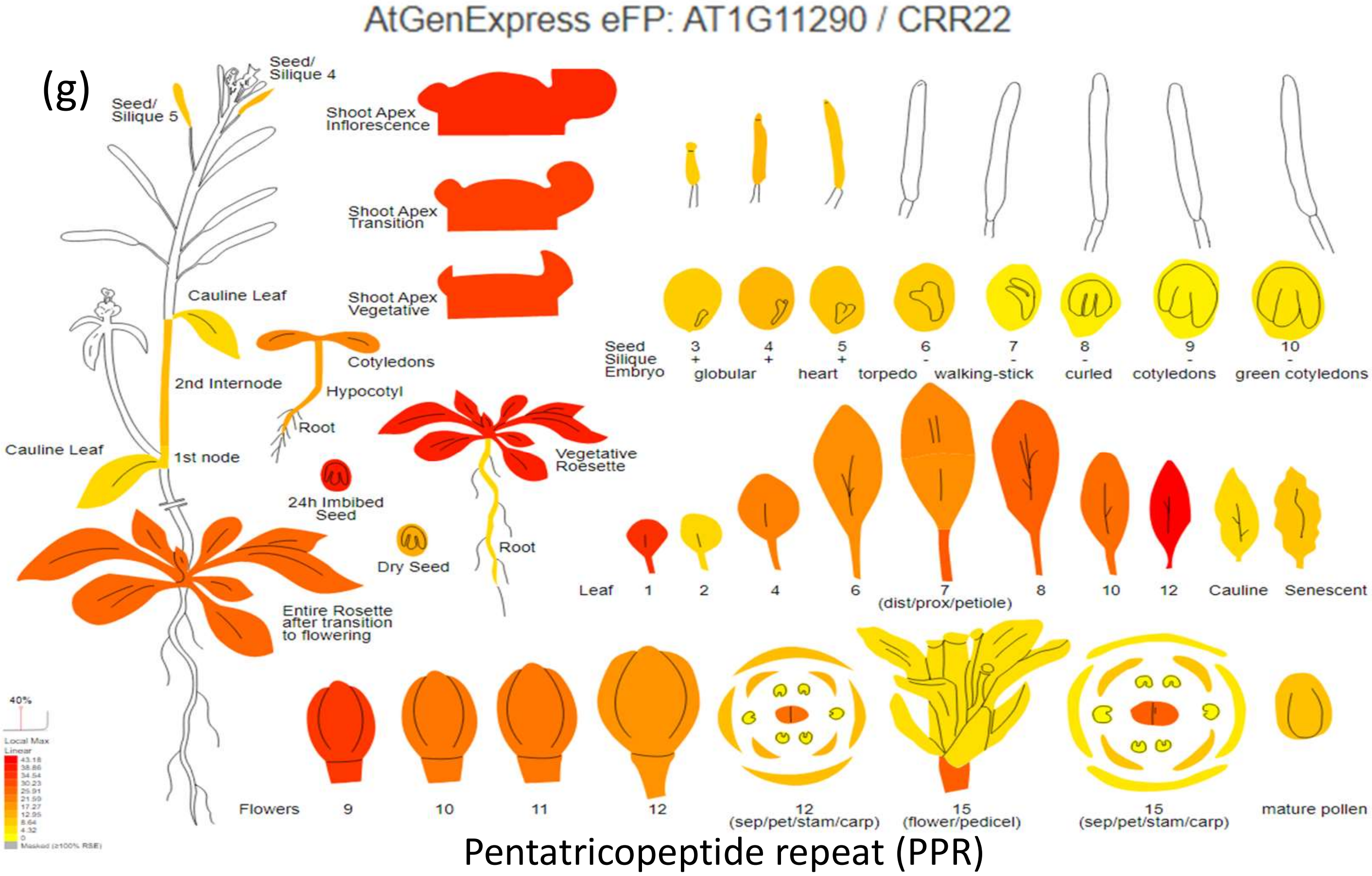

Pentatricopeptide repeat (PPR)

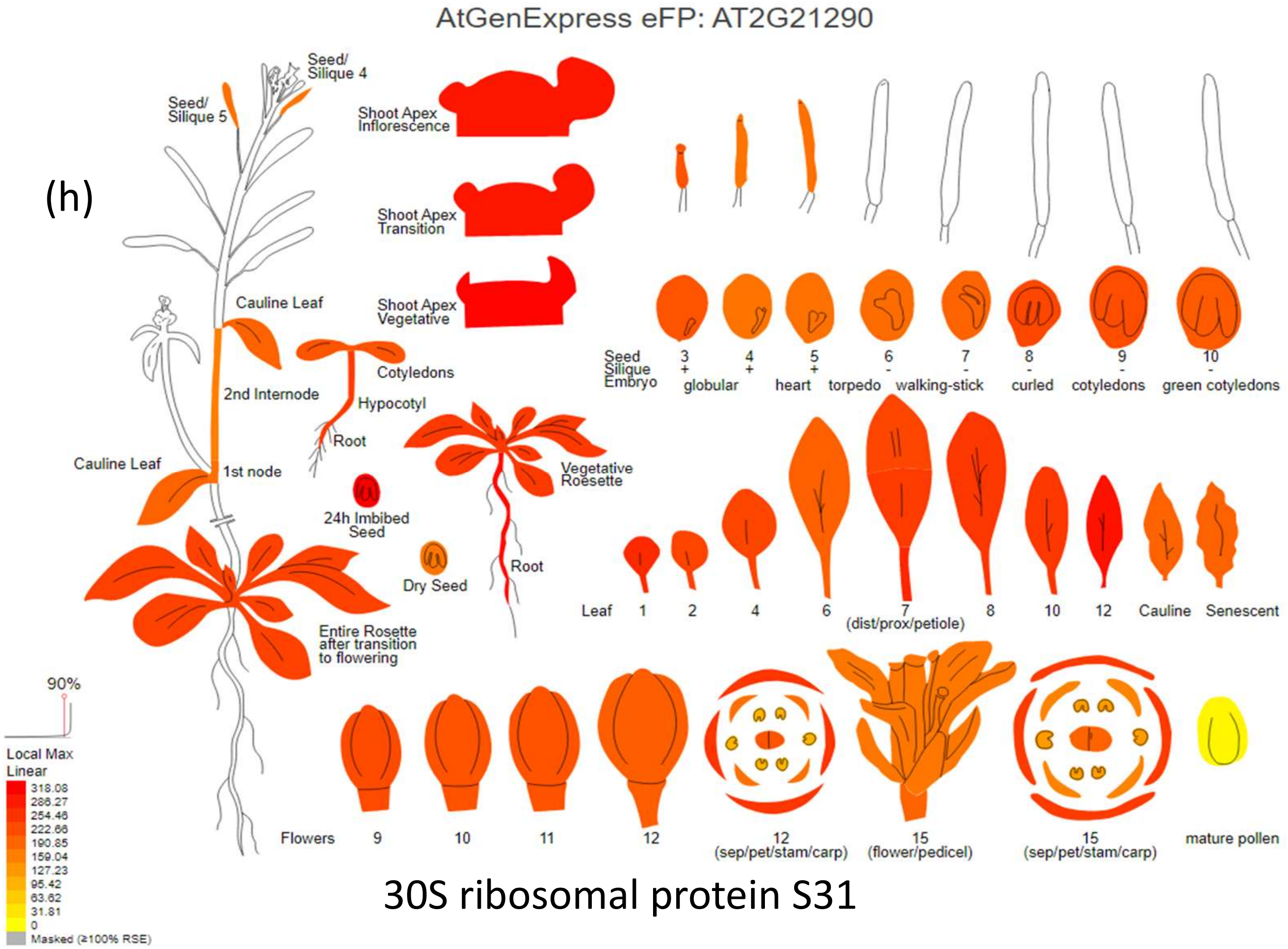

30S ribosomal protein S31

Figure S7. (g) Pentatricopeptide repeat (PPR) superfamily protein gene expression for disease resistance in leaves on chr. 5. (h) 30S ribosomal protein S31 gene expression for disease resistance in leaves on chr. 9.

Leaf area

(i)

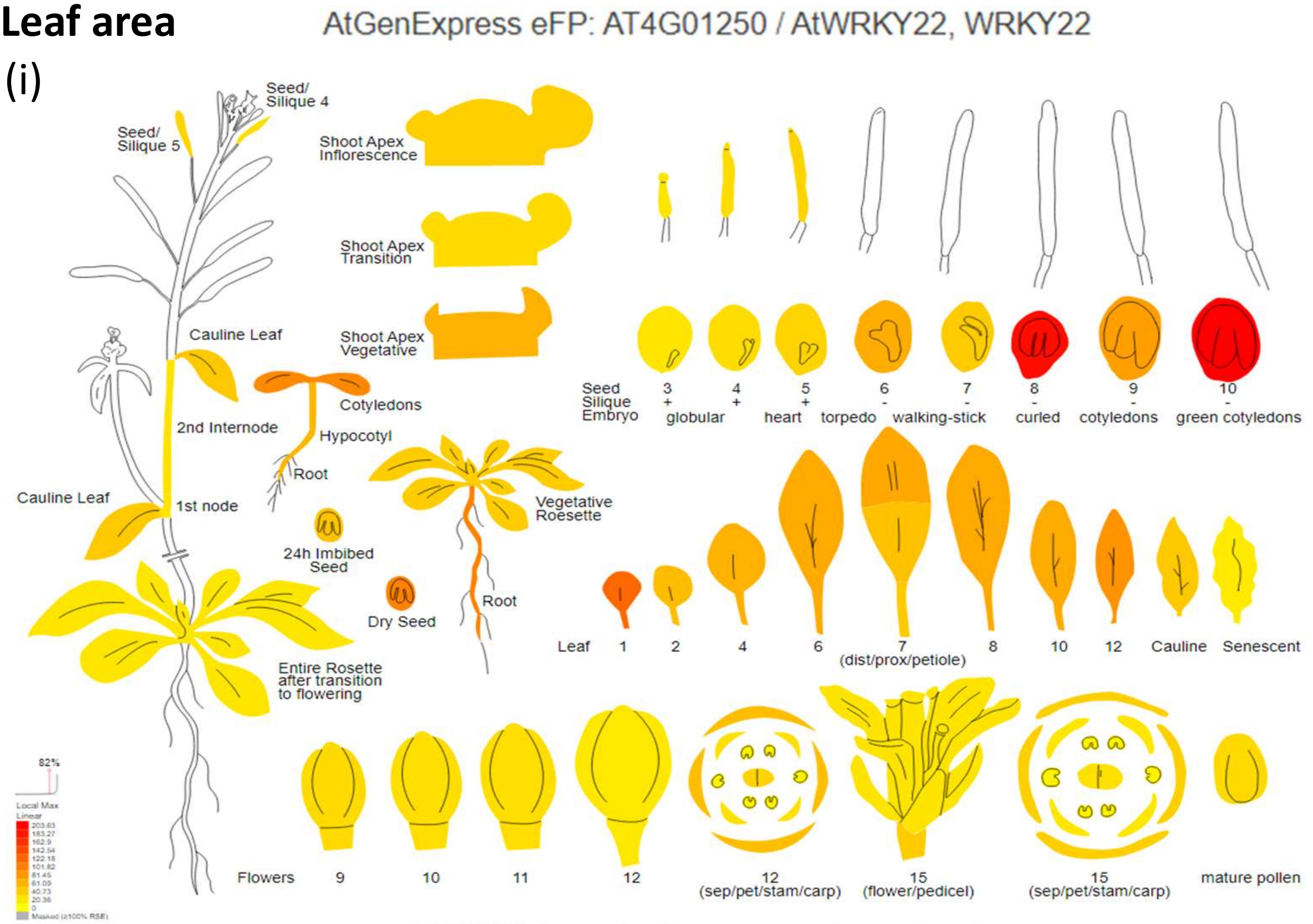

WRKY family transcription factor

Trichomes

(j)

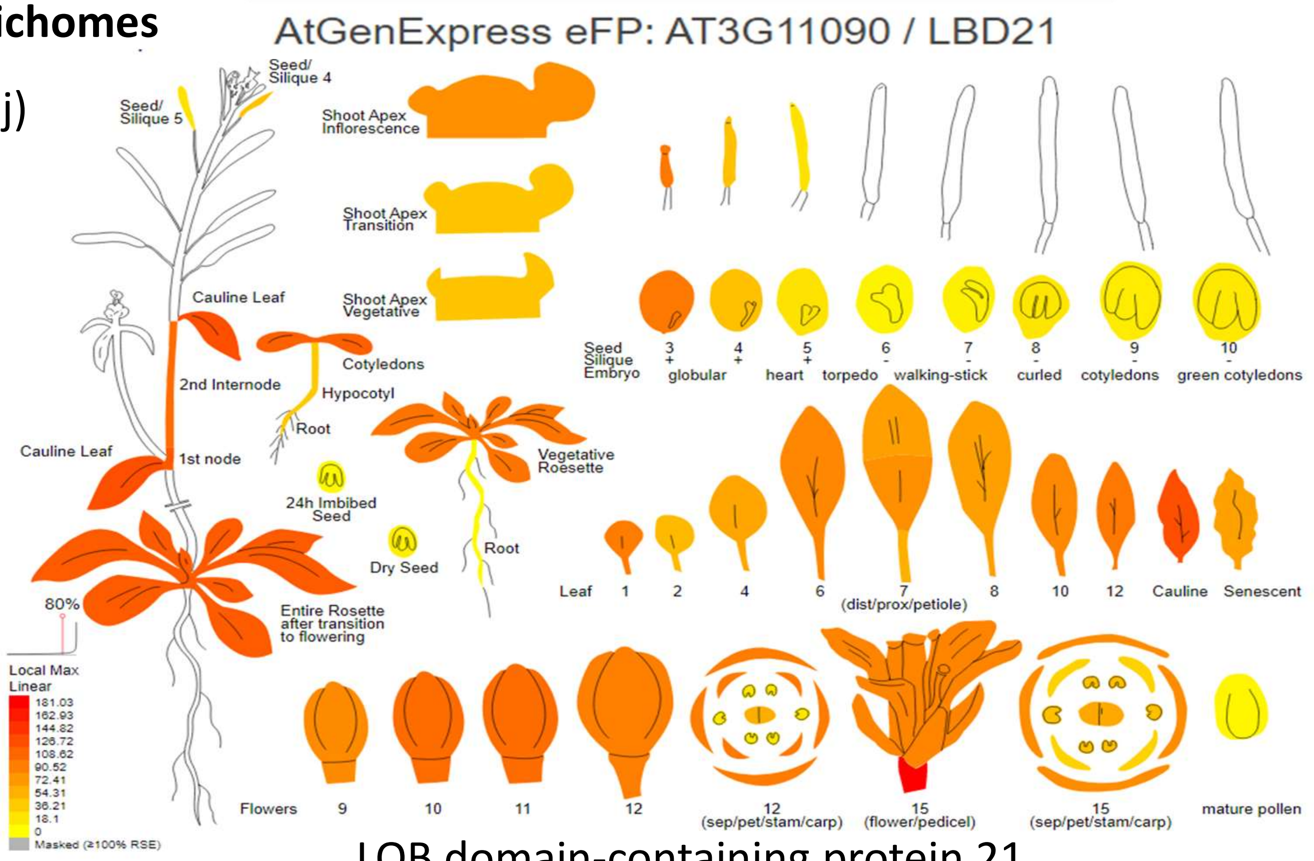

LOB domain-containing protein 21

Figure S7. (i) WRKY family transcription factor gene expression in leaf on chr. 3. **(j)** LOB domain-containing protein 21 gene expression in trichomes on chr. 6.

SPAD

(k)

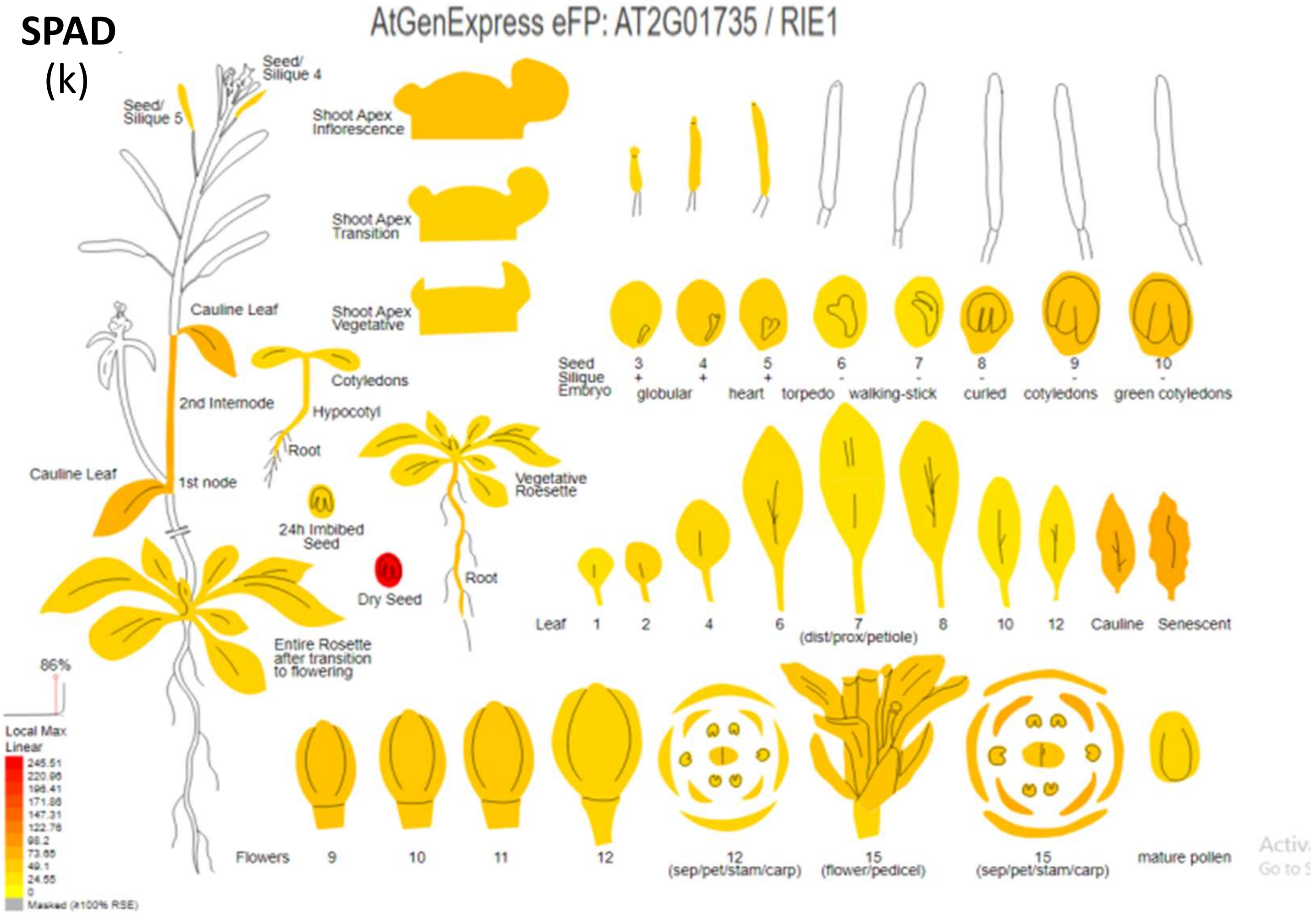

E3 ubiquitin protein ligase RIE1

Figure S7. (K) E3 ubiquitin protein ligase RIE1 gene expression in for chlorophyll content/ SPAD value on chr. 11.
